# Blended SORT-IT for operational research capacity building: the model, its successes and challenges

**DOI:** 10.1080/16549716.2018.1469215

**Published:** 2018-05-10

**Authors:** Tom Decroo, Rafael Van den Bergh, Ajay M. V. Kumar, Rony Zachariah, Erin Schillberg, Philip Owiti, Wilma van den Boogaard, Guido Benedetti, Safieh Shah, Engy Ali, Anthony D. Harries, Anthony J. Reid

**Affiliations:** a Médecins Sans Frontières (MSF), Medical Department, Operational Centre Brussels, MSF Luxembourg, Luxembourg; b Department of Clinical Sciences, Institute of Tropical Medicine, Antwerp, Belgium; c International Union Against Tuberculosis and Lung Disease (The Union), South-East Asia Office, New Delhi, India; d Special Programme for Research and Training in Tropical Diseases (TDR), World Health Organization, Geneva, Switzerland; e Academic Model Providing Access to Healthcare (AMPATH), Eldoret, Kenya; f International Union against Tuberculosis and Lung Disease, Paris, France; g Department of Clinical Research, London School of Hygiene and Tropical Medicine, London, UK

**Keywords:** Operational research, capacity building, blended learning, mentorship, education, training, SORT-IT

## Abstract

The Structured Operational Research Training Initiative (SORT-IT) has been shown to be very effective in strengthening capacity for conducting operational research, publishing in scientific journals and fostering policy and practice change. The ‘classic’ model includes three face-to-face modules during which, respectively, a study protocol, a data analysis plan, and a manuscript are elaborated. Meanwhile, the lectures of the SORT-IT are available online as YouTube videos. Given the availability of this online material and the experiences with online mentorship of the faculty, we piloted a first blended distance/residential SORT-IT. To inform future implementers of our experience with blended operational research courses, we summarize the model, successes, and challenges of this approach in this perspective paper. The blended SORT-IT consisted of an online phase, covering modules 1 and 2, followed by a face-to-face writing module 3. Four out of six participants successfully completed the course, and submitted a manuscript to a peer-reviewed journal within four weeks of completing module 3. A blended approach may make the SORT-IT course more accessible to future participants and may favour the adoption of the course by other institutions, such as national Ministries of Health.

## Background

Operational research (OR) is increasingly used to study how interventions with a demonstrated efficacy can be applied in an effective manner in programmatic settings, especially when the disease burden is high, resources are scarce, and difficult choices need to be made.^1^ The Structured Operational Research Training Initiative (SORT-IT), is a Global Partnership Initiative coordinated by the Special Programme for Research and Training in Tropical Diseases at the World Health Organization (WHO-TDR) since 2012 and implemented with partners. The SORT-IT is an output-based course, whereby public health programme staff are mentored in OR while working on their own research studies. About 90% of all participants have achieved the pre-defined milestone of completing and submitting a scientific paper to a peer-reviewed Journal [1,2].

SORT-IT consists of 3 one-week residential modules that are spread over 9 to 12 months. To successfully complete the course, participants have to achieve four milestones, which include submission of: (1) an OR protocol at the end of module 1; (2) data collection tools and dummy tables after module 2; (3) a complete dataset before module 3; and (4) proof that the study was submitted to a peer-reviewed journal within four weeks of the end of module 3 [3]. The course uses the same standardized lecture material for the many courses that have been run in Africa, Asia, South-Pacific, Western-Europe, Eastern-Europe, and South America. The lectures have been recorded, and are currently available on YouTube (https://www.youtube.com/channel/UC9ZRuVhbrxJm5xAjUHwo6Hw).

To join the classic SORT-IT course, participants and mentors should be available to join three face-to-face modules of one week each. However, potential participants with many competing responsibilities may have difficulties in blocking off three weeks in their agenda. Moreover, face-to-face modules are more costly for the organizer, due to travel and accommodation expenses, and visa issues may preclude worthy candidates from attending global/regional courses. The costs may also limit the adoption of the SORT-IT course by other institutions, such as national Ministries of Health, who probably could benefit most from OR on programme data.

Therefore, a blended approach of distance and residential training, named ‘blended SORT-IT’, was proposed to explore whether these barriers could be overcome, while safeguarding the course’s core principles. The SORT-IT staff agreed to provide modules 1 and 2 online. We anticipated a weekly student investment time of eight hours, with one lecture and one assignment per week. But we agreed that module 3 should remain face-to-face as close contact between participants and mentors and dedicated time was thought to be critical for developing and finalizing a scientific paper. The first blended SORT-IT course was piloted by Médecins Sans Frontières (MSF) Luxembourg between October 2015 and April 2016. Six participants associated with MSF were enrolled. As in the classic SORT-IT, mentors had complementary skills, including statistical and editorial experience. We aimed at including participants with a basic knowledge of managing a database and performing descriptive statistics, given that the blended SORT-IT included fewer lectures on these aspects.

In this short communication we report how the classic SORT-IT course was adapted to fit a blended approach. In addition, we report the output, challenges encountered, and recommendations for future blended SORT-IT courses. The sources of data that are presented here include the documents that were used during the course implementation and the evaluations by the participants and mentors.

### The model

Table 1 shows how the building blocks of the classic SORT-IT course were translated to a blended version. Modules 1 and 2 of the classic SORT-IT were provided during the online phase using the same structured approach. Participants and their mentors navigated through the different steps: first, they identified a research question; second, they developed the Introduction, Methods, and Ethics sections of a study protocol; and finally, they created dummy tables and conducted a draft data analysis. Input consisted of lectures shown through YouTube videos and weekly assignments. The course content of modules 1 and 2 of the blended SORT-IT was similar to the classic SORT-IT, except for the lectures on EpiData. As we expected participants to already have an existing database, lectures on the use of EpiData were not included. The face-to-face module of the blended SORT-IT was similar to the module 3 of the classic SORT-IT.

**Table 1.** Designed outline of the blended SORT-IT, compared to the classic SORT-IT.

	SORT-IT – classic	Blended SORT-IT ^(a)^
Module 1	Provided lectures include Introduction to OR and SORT-IT Examples of OR Literature review Study designs used in OR Statistics Research ethics principles Citation and referencing Authorship rules After the lectures, participants meet with their mentors, and develop stepwise the protocol, whereby every new version elaborated by the participant is revised by the mentor. Different sections (Objectives, Introduction, Methods) are presented and commented upon during different plenary sessions.	Step 1: YouTube videos provide an introduction to OR and SORT-IT. As assignment, participants describe barriers they anticipate facing when engaging with their study. Step 2: YouTube videos show examples of OR. Participants develop the OR question with support from their mentors. Step 3: A YouTube video shows how to conduct a literature review. An example of an Introduction from another study protocol is shared. Participants develop the Introduction of their study protocol with support from their mentors. Step 4: YouTube videos explain study designs and terms often used in OR, basic statistics, and an example of a study protocol. Participants develop the Methods of their study protocols with support from their mentors. Step 5: YouTube videos explain research ethics, citation and referencing, and authorship rules. Participants add an Ethics section, edit the references, and add authors to the protocol, and then finalize their study protocols with support from their mentors. The protocol is then submitted for ethics review.
Module 2	Provided lectures include EpiData software for encoding and analysing data Data analysis plan After the lectures, participants meet with their mentors, and develop the EpiData database, data analysis plan, and dummy tables/graphs.	Step 6: A Word document explains the concept of dummy tables and gives an example. Participants develop dummy tables with support from their mentors. Step 7: Participants perform descriptive statistics using their study data with support from their mentors. A Word document is shared summarizing frequently used statistics.
Module 3	Provided lectures include Presentation of tables and figures What do editors expect? Example of an introduction and method Example of results Example of a discussion Finalization of the manuscript: title, authors, affiliations, references After the lectures, participants meet with their mentors, and develop stepwise the manuscript, whereby every new version elaborated by the participant is revised by the mentor. Different sections (Introduction, Methods, Results, Discussion) are presented and commented upon during different plenary sessions.	The writing module is similar for both the classic and blended SORT-IT Participants meet as a group for the first time for this writing module, and spend the first two days on summarizing data and presenting the data in tables and figures. After a resting day, the subsequent five days are similar to what is provided in module 3 of the classic SORT-IT.

SORT-IT: Structured Operational Research Training Initiative.

^(a)^ YouTube videos used during blended SORT-IT: https://www.youtube.com/channel/UC9ZRuVhbrxJm5xAjUHwo6Hw.

**Figure 1.**
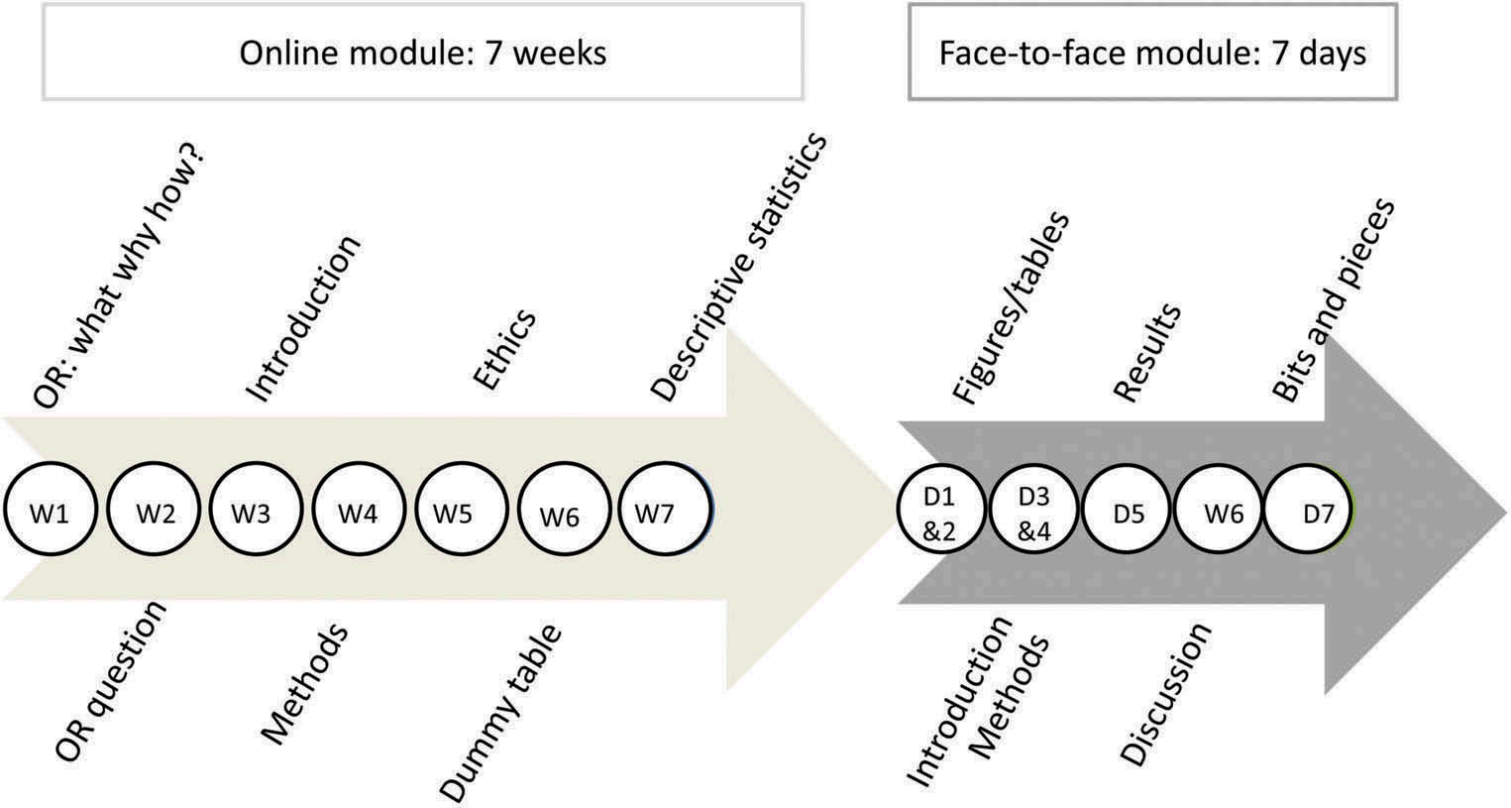
Timeline of the online and face-to-face module of the blended SORT-IT. W: week; D: day

Table 2 shows how milestones were slightly adapted to fit the blended SORT-IT. Figure 1 shows the timeline and content of the online and face-to-face modules.

**Table 2.** Milestones for the blended SORT-IT, compared to the classic SORT-IT.

	SORT-IT – classic	Blended SORT- IT
Milestone 1	**Three weeks after the end of module 1**: Submission of the research protocol and the completed ethics form to the module director, course coordinator, and respective ethics committee.	**After finishing step 5**: Submission of the research protocol and the completed ethics form to the mentor, course coordinator, and respective ethics committee.
Milestone 2	**Two weeks after the end of module 2**: Submission of data documentation sheet, EpiData triplet files (qes, rec, and chk files) and dummy tables to the module director and course coordinator.	**After finishing step 5**: Submission of dummy tables/graphs to the mentor and course coordinator.
Milestone 3	**Six weeks prior to the start of module 3**: Submission of completed datasets and draft analysis to the module 2 facilitators and course coordinator.	**Two weeks prior to the start of module 3**: Submission of completed datasets and draft analysis to the mentor and course coordinator
Milestone 4	**Four weeks after the end of module 3**: Submission of a paper to a peer-reviewed journal: copy of the submitted paper and electronic confirmation of receipt of the submitted paper by the journal to be sent to both the module director and the course coordinator.	**Four weeks after the end of module 3**: Submission of a paper to a peer-reviewed journal: copy of the submitted paper and electronic confirmation of receipt of the submitted paper by the journal to be sent to both the mentor and the course coordinator

SORT-IT: Structured Operational Research Training Initiative

### Outputs

Table 3 shows how five participants who joined the face-to-face writing module scored several key aspects of the course at the end of module 3. Participants were especially satisfied with the availability and support of mentors during the online module:‘My mentors were very supportive and helpful in clarifying the objectives and developing the protocol (that was an important educational process). It was a “back and forth” process in which they were both quick and precise.’ Overall, participants were satisfied: ‘I learnt a lot from my colleagues and mentors. The presentations were very useful and straight to the point and practical.’ This was reflected in the score of 80.8%. However, participants felt they could have been better prepared during the online module. The use of a central online repository could have improved the oversight during the online module. Moreover, the experience during the face-to-face writing module was rated as very intense.

**Table 3.** Score for six indicators, by five participants, at the end of module 3 of the blended SORT-IT.

	Excellent (4 points)	Good (3 points)	Average (2 points)	Insufficient (1 point)	Very poor (0 points)	Score on 20 points ^$^	%
Online course materials	1	4	0	0	0	16	80
Scheduling of the online course	1	4	0	0	0	16	80
Mentor support during the online part of the course	2	3	0	0	0	17	85
Level of preparation at the end of the online part of the course, for joining the writing module?	1	3	0	1	0	14	70
Overall content of the writing module	5	0	0	0	0	20	100
Overall format of the writing module (duration, schedule)	1	3	0	1	0	14	70
**Total**						**16.2**	**80.8**

^$^Calculated as the sum of the products of the number of participants and the value that corresponds with each score.

Table 4 summarizes the outputs from the course. One out of six participants dropped out before milestone 1, as he left for a mission and was scheduled to join another SORT-IT course. The remaining five participants managed to develop a protocol (milestone 1), dummy tables (milestone 2), submitted a dataset (milestone 3), and were able to join the writing module 3, although most submitted their datasets late. All participants in the face-to-face writing module completed a draft manuscript at the end of the module. Four participants achieved the fourth and final milestone – submitting their manuscript to a peer-reviewed journal before the deadline. One participant did not submit a manuscript and thus failed. Her manuscript later served as a dissertation, submitted for obtaining a degree in Master of Public Health. One participant achieved milestone 4, but had to withdraw the paper after submission, because one partner of the multi-centric sites did not agree with the publication of the data. Three manuscripts were published by the end of September 2017, 17 months after completing the course.

**Table 4.** Output of the MSF Luxembourg blended SORT-IT pilot, held between October 2015 and April 2016.

	Profile	Reason for joining the blended SORT-IT	Research subject	Milestone 1	Milestone 2	Milestone 3	Final draft of a manuscript at the end of the writing module	Milestone 4	Publication status, by 30/09/2017 (17 months after module 3)
Participant 1	WASH	No call was open when the participant showed interest	Ebola Virus Disease	Not completed	Dropped out	Dropped out	Dropped out	Dropped out	Dropped out
Participant 2	MD	Availability didn’t match the classic SORT-IT	Ebola Virus Disease	Completed	Completed	Dataset submission was delayed	Completed	Not completed	Not published
Participant 3	MD	Availability didn’t match the classic SORT-IT	Mental Health	Completed	Completed	Dataset submission was delayed	Completed	Completed	Published
Participant 4	Epidemiologist	Not selected for a previous SORT-IT	Vaccination	Completed	Completed	Dataset submission was delayed	Completed	Completed	Not published
Participant 5	Physiotherapist	Not selected for a previous SORT-IT	Physiotherapy	Completed	Completed	Completed	Completed	Completed	Published
Participant 6	MD	No call was open when the participant showed interest	Viral load monitoring in people living with HIV	Completed	Completed	Dataset submission was delayed	Completed	Completed	Published

MSF-OCB: Médecins sans Frontières – Operational Centre Brussels; WASH: water, sanitation and hygiene; MD: medical doctor; DRC: Democratic Republic of Congo; SORT-IT: Structured Operational Research Training Initiative.

### Challenges

Table 5 summarizes the challenges encountered during the course, and shows recommendations. The main challenges were related to obtaining and preparing the data before arriving at module 3. Moreover, as some participants were less experienced than expected in managing and analysing data, most of their data cleaning and analysis had to be done during the face-to-face module. This put a lot of pressure on both the participants and mentors. Therefore, future organizers of blended SORT-IT courses should consider placing more emphasis on data analysis during the distance phase or extending the face-to-face writing module by an extra day. Alternatively, they could take up a short course on statistics prior to enrolment on a SORT-IT course.

**Table 5.** Challenges, solutions applied during the blended SORT-IT, and recommendations for future blended SORT-IT courses.

	Challenges	Solutions applied during the implementation of the blended SORT-IT	Recommendations for future blended SORT-IT courses
Selection criteria	The participants had varying degrees of comfort with data analysis. This course was intended for those with adequate analysis skills, and not all participants possessed them.	Mentors were intensively involved in data cleaning and analyses, especially during the first three days of the writing module.	Provide the course to participants with a basic experience in operational research and data analysis.
Online course platform	Emails were used, but were difficult to track in the inboxes of both mentors and participants. As a result, it was not always easy to follow.	To engage participants and mentors more emails than strictly necessary were sent.	Consider Dropbox for the submission of documents, and subsequent versions of study protocols and dummy tables.
Online course material	The YouTube videos were used and were seen as a positive influence. However, the subjects of the presented examples should cover other themes than HIV & TB only. Students felt they could have benefitted from more lectures on statistics and software that can be used for data capture and data analysis.	Not applicable	Consider developing YouTube videos covering a broader range of subjects.
Availability of participants and mentors	Mis-match of availability of the participants and mentors delayed development of the study protocols and submission of datasets and dummy tables. Although regular updates and suggested deadlines were communicated, often the participants were not able to meet them.	Deadlines were extended, and applied flexibly	The availability of mentors could be organized differently during the online phase. Mentors could take turns, and be available for a specific task involving all participants during a short period, instead of being continuously available for a long period for one participant. Apply deadlines more strictly.
Preparedness for module 3, the writing module	Most of the participants arrived at the writing module with less-well-developed datasets than usual for SORT- IT courses. This put extra pressure on participants and mentors to complete the write-up.	Given the delayed submission of datasets, the usual first two days of table development spilled over into the third (rest) day. Fortunately, having that extra day helped to have a product ready for writing on the fourth day.	Foresee three days on tables and figures. Ensure that at least half of the mentors are experienced in data cleaning and analyses.

SORT-IT: Structured Operational Research Training Initiative.

## Discussion

Between October 2015 and April 2016, the operational research course, SORT-IT, was adapted to a blended format consisting of an online phase covering modules 1 and 2, followed by a face-to-face writing module 3. Four out of six participants successfully completed the course, and submitted a manuscript to a peer-reviewed journal within four weeks of completing module 3 (milestone 4).

To assess the performance of SORT-IT courses, ambitious 80-80-80-80 targets are employed. The following four indicators should have a score of 80% or more: (1) the proportion of the total course marks given by the participants; (2) the proportion of enrolled participants who achieved milestone 4; (3) the proportion of submitted papers that were accepted, in press or published within 18 months of first submission; (4) the proportion of participants who completed their projects and provided follow-up information within 18 months on whether their projects informed policy and practice change [3].

For the evaluation of this course we assessed these indicators. The overall score from the five participants who joined the writing module was 80.8%. But only four out of six (66.7%) participants completed milestone 4 and three out of four submitted manuscripts were published (milestone 3). Hence, the 80-80-80-80 targets were not met, which contrasts with the success of the classic SORT-IT [1,2]. On the other hand, the proportion who successfully published is higher than that of some other OR courses [4]. The output could be improved if lessons learnt from this pilot experience are applied. Future Blended SORT-IT courses may consider a more rigorous selection of participants, taking into consideration data management skills. The pilot Blended SORT-IT was organized for MSF-affiliated participants only, and they were not selected out of a large group of candidates, as is the case for the classic version [3]. To maximize the chance of identifying candidates with the desired profile and thus a successful completion of the SORT-IT course, it may be desirable to offer the possibility of applying to a wider audience [3].

On the other hand, we think that the course achieved one of its important aims: capacity building in OR. The five participants who completed the course developed a scientific manuscript, step by step, addressing the research question they brought to the course. During this process they acquired skills to identify an OR question and write up a protocol and a manuscript. Previous studies have shown that after the course about half of the alumni complete a new research project and about one in three participants joins a SORT IT course as a facilitator, which shows a steadily increasing capacity for OR [5–7].

In conclusion, the blended SORT-IT was conceived as a complementary approach to building OR capacity. It may be suitable for participants with a basic knowledge of statistics, and a commitment to achieving the goals of the course, but who are unable to attend the classic SORT-IT courses due to time or availability constraints.

## Paper context

The Structured Operational Research Training Initiative (SORT-IT) has been shown to be very effective in strengthening capacity for conducting operational research, publishing in scientific journals and fostering policy and practice change. A blended approach of distance and residential training may make the SORT-IT course more accessible to future participants and may favour the adoption of the course by other institutions, such as national Ministries of Health.
